# Increased PACAP- and DβH-Positive Hepatic Nerve Fibers after Bisphenol A Exposure

**DOI:** 10.3390/toxics9050110

**Published:** 2021-05-18

**Authors:** Michael Thoene, Liliana Rytel, Ewa Dzika, Joanna Wojtkiewicz

**Affiliations:** 1 Department of Medical Biology, Faculty of Health Sciences, University of Warmia and Mazury in Olsztyn, Żołnierska 14C Str., 10-561 Olsztyn, Poland; e.dzika@uwm.edu.pl; 2 Department of Internal Medicine and Clinic, Faculty of Veterinary Medicine, University of Warmia and Mazury, Oczapowskiego Str 15, 10-718 Olsztyn, Poland; Liliana.Rytel@uwm.edu.pl; 3 Department of Pathophysiology, Faculty of Medical Sciences, University of Warmia and Mazury, 10-718 Olsztyn, Poland; Joanna.Wojtkiewicz@uwm.edu.pl

**Keywords:** bisphenol A, behavioral/psychological disorders, immunofluorescence technique, sympathetic nervous system, childhood exposure to BPA

## Abstract

Bisphenol A (BPA) is an endocrine-disrupting compound (EDC) that can be found nearly everywhere in our polluted world. BPA has been correlated with pathophysiologies that include psychological disorders, especially in children. This study used juvenile porcine models to investigate the effects of BPA on the liver of developing vertebrates in order to determine the effects of BPA on innervated hepatic samples with the use of double-labeled immunofluorescence. This study specifically investigated the sympathetic nervous system (SNS) colocalized with a specific neural marker (PACAP) that has previously been correlated with specific pathophysiologies in the literature. In the liver, it was observed that there were significantly increased nerve fibers in the SNS colocalized with the neural marker PACAP after exposure to BPA at concentrations considered to be “safe” with the same or more profound effects at higher exposure levels. The implications of childhood exposure to BPA are then discussed with regard to several correlation studies that have linked BPA exposure to behavioral/psychological disorders. It is possible that BPA exposure in childhood may upregulate the SNS and PACAP levels, thereby contributing to the correlations in the literature.

## 1. Introduction

Bisphenol A (BPA) is a very common plastic polymer that has many uses, especially in the food industry. It has been used for decades as an epoxy resin to line canned foods and drinks, it is used in food packaging and food storage containers, and in some cities, it has even been used to line pipes that carry drinking water to households. Therefore, BPA has a very high toxic profile [1,2,3]. Products containing BPA and other bisphenols were originally considered to be safe, but recently it has been found that these bisphenol compounds act very much like estrogen in the body, and very small amounts can cause endocrine disruption. As an EDC (endocrine-disrupting compound), it is suspected of disrupting several endocrine system signaling pathways, including androgen receptors and thyroid hormone receptors even at nanogram concentrations [3]. This effect may be especially evident in developing vertebrates. The aim of this paper was to investigate the effects of BPA on innervated hepatic tissue taken from young porcine animal models at 8 weeks of age. Since much of the correlation literature points to the effects of bisphenol on developing human children, developing pigs were used in this study to simulate what may be happening in human children. This particular study used a marker for the sympathetic nervous system (DβH) colocalized with the neuronal marker PACAP in an effort to observe increased or decreased positive nerve fibers in the developing liver at low and high levels of BPA exposure, as compared to controls. In this communication, PACAP has the exclusive focus since our previously published work has already reported on several of the other neural markers within the sympathetic nervous system [4].

All of the involuntary systems of the body are controlled by the autonomic nervous system (ANS). It is split into two subtypes: the parasympathetic nervous system (PNS) and the sympathetic nervous system (SNS). The PNS controls the “rest and digest” functions of metabolism, while the SNS controls the “fight or flight” response. The SNS tends to have short neuronal pathways and very fast transduction times, which makes sense for a system designed to control a threat response. Mainly, the SNS controls levels of catecholamines, such as epinephrine, norepinephrine, and dopamine [5]. This study investigated the SNS of porcine liver for DβH and PACAP in pigs at the age of 8 weeks. These neuronal markers have previously been described as “gut–brain” peptides [6]. Since most nutrients are absorbed in the small intestine and are quickly sent to the liver via the hepatic portal vein, this is relevant. Therefore, if dietary BPA increases or decreases the number of DβH- or PACAP-positive nerve fibers, then the amount of BPA in the diet of the experimental models used in this study should quickly affect the innervation of the developing juvenile liver, as compared to control groups.

Any statistically significant changes in the number of DβH- or PACAP-positive nerve fibers may be compared to previous correlation studies in the literature. This study used immunofluorescence assay techniques to colocalize dopamine beta-hydroxylase (DβH), a marker specific for the SNS, and pituitary adenylate cyclase-activating polypeptide (PACAP). DβH is a catecholamine synthesis enzyme responsible for the production of adrenaline, as well as other related compounds. Increased levels of PACAP have been correlated with psychological disorders. Therefore, if either or both of these markers show increases after BPA administration, it could possibly indicate that BPA compounds are adversely affecting innervation patterns in children that could have observable clinical effects.

## 2. Materials and Methods

The present study was conducted on 15 immature sows of the Large White Polish breed at the age of 8 weeks and about 18–20 kg body weight. The animals were kept in typical laboratory conditions that had been adapted for this animal species. The experiment was carried out in compliance with the instructions of the Local Ethical Committee for Experiments on Animals in Olsztyn (Poland) (decision number 28/2013).

The animals were divided randomly into three groups after a 3-day adaptive period: (1) control group—placebo (empty gelatin capsules for 28 days during feeding); (2) experimental group I (received BPA capsules at a dose acceptable under European legislation—0.05 mg (50 μg)/kg bw/day); (3) experimental group II (received BPA capsules at a dose 10 times higher than the acceptable level—0.5 mg/kg bw/day). All pigs were weighed every four days before the morning feeding. This was done to determine their body weight and properly calculate the BPA dosage.

After administering BPA for 28 days, the pigs were premedicated using Stressnil (Janssen, Belgium, 75 μL/kg of body weight, i.m.). After approximately 30 min, the animals were euthanized with a sodium thiopental overdose (Thiopental, Sandoz, Kundl-Rakúsko, Austria, i.v.) and transcardially perfused with 4% buffered paraformaldehyde. Tissue samples were collected from all animals. Liver tissue sections were fixed in the paraformaldehyde solution mentioned above, rinsed for three days in phosphate buffer, and stored at 4 °C in 18% sucrose. After a minimum of two weeks, the liver fragments were frozen at −23 °C. They were then sliced using a microtome (Microm, HM 525, Walldorf, Germany) into 10 μm-thick sections and were subjected to a routine double-label immunofluorescence technique. This technique has been previously described by Gonkowski [7,8] and Wojtkiewicz [6,9]. The following is a brief description of the above-mentioned method: 45 min of drying followed by incubation with a blocking solution that included 0.1% bovine serum albumin, 10% normal goat serum, 0.01% NaN_3_, thimerosal and Triton X-100 in PBS for 1 h; incubation overnight with a mixture of two antibodies produced in different species (both were primary) and raised against dopamine beta-hydroxylase (DβH) and PACAP; and one hour of incubation using species-specific antisera that had been FITC- or biotin-conjugated. This was visualized with a streptavidin–CY3 complex. Table 1 specifies which primary and secondary antibodies were used in the present study. Between each of the stages, the sections were rinsed with PBS (3 × 10 min, pH 7.4).

Standard controls were conducted for the specificity of “primary” antibodies during this study, including the preabsorption of each particular antiserum with its appropriate antigen. Moreover, “omission” and “replacement” tests were performed that completely eliminated the immunofluorescence signals.

Two methods of evaluating the colocalization of DBH with PACAP were used. The first method consisted of determining the number of nerve fibers found in the field of view during microscopic examination of the three animal groups. The second method consisted of determining what percentage of all DBH-LI nerve fibers were neurally immunoreactive with PACAP. To this end, DBH-positive nerve fibers in hepatic tissues were examined for immunoreactivity with regard to PACAP. DBH-positive nerve fibers were considered as representing 100% for all combinations. This method was performed to determine what percentage of DBH-positive nerve fibers were immunoreactive with PACAP. Two independent investigators evaluated the immune-positive nerve fibers by counting the nerves. An Olympus BX51 microscope equipped with epifluorescence and appropriate filter sets was used to visualize the double-labeled nerve fibers. The results that were recorded were pooled and tabulated as a mean ± SEM. In order to prevent the double counting of the same neuronal cells, all of the examined sections in this study were located at least 100 µm apart. Student’s *t*-test was used for statistical analysis (Graphpad Prism v. 6.0; GraphPad Software Inc., San Diego, CA, USA). Statistical significance for all differences was set at *p* ≤ 0.05.

## 3. Results

### 3.1. Significantly Increased Density of SNS Nerve Fibers

In the innervated hepatic cells, there was an increased number of DβH+ nerve fibers observed after both low and high doses of dietary BPA as compared to the control samples. Even at suggested safe levels, DβH was elevated. Compared to the control group, the number of DβH+ nerve fibers was increased by 48.6%. Furthermore, when animals were exposed to 10 times above the recommended safe level of BPA, the number of DβH+ nerve fibers was 63.7% higher than the control samples. These results were statistically significant. Since DβH is generally accepted to be a marker for the SNS, these results suggest an upregulation of the SNS after either low or high BPA exposure.

### 3.2. Significantly Increased Density of PACAP+/DβH+ Nerve Fibers Colocalized in the SNS

**Figure 1:**
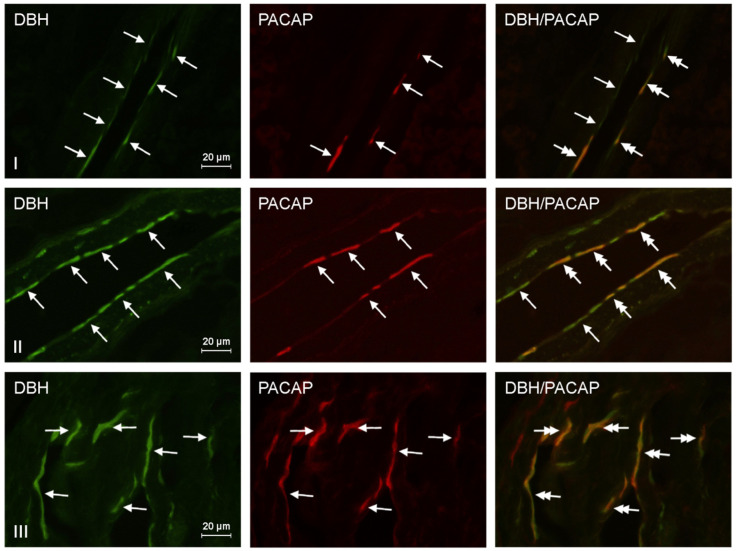
Representative images of the immunocytochemical localization of DβH- and PACAP-immunoreactive nerve fibers in intrahepatic sympathetic nerves (arrows) of the control group (CTRL, (**I**)), experimental group 1 (E1, low dose of bisphenol A; (**II**)), and experimental group 2 (E2, high dose of bisphenol A, (**III**)). DβH or PACAP-IR nerve fibers are indicated with arrows; DβH and PACAP-IR nerve fibers are indicated with double-headed arrows; scale bars in all figures represent 20 μm.

Compared to the control group, the number of PACAP+/DβH+ nerve fibers observed after exposure to suggested safe levels of BPA was increased by 166.7%. Moreover, when animals were exposed to 10 times above the recommended safe level of BPA, the PACAP+/DβH+ nerve fiber count was 135.6% higher than the control. All results showed statistical significance. The above-mentioned results are presented in Table 2. Representative images of DβH/PACAP immunocytochemical colocalization are shown in Figure 1.

## 4. Discussion

Dopamine beta-hydroxylase has been known as an enzyme important for proper neural functioning for over fifty years. Patients who are born without a functional gene encoding for DβH have severe health problems characterized by increased dopamine levels and the complete absence of epinephrine and norepinephrine in the blood plasma [10]. DβH is a membrane-bound copper-conjugated oxygenase that catalyzes the conversion of dopamine to norepinephrine. DβH also uses ascorbate as a cofactor. The lack of this enzyme in humans is quite rare but allowed clinical scientists to characterize this protein quite early on. The lack of a functional DβH enzyme will not allow the production of adrenaline from dopamine, which is essential for the sympathetic nervous system to function properly. DβH is expressed in both the central nervous system and peripheral nervous system in noradrenergic nerve terminals. It is also expressed in chromaffin cells located inside of the adrenal medulla, which is responsible for catecholamine production [11,12]. Therefore, DβH is crucial for the initiation of the sympathetic nervous system and the resulting “fight or flight” response. For this reason, DβH is commonly used as a marker for the SNS, and this study used DβH to investigate the hepatic nerve fibers of the sympathetic nervous system after exposure to varying levels of BPA.

DβH has been visualized quite often via immunohistochemistry using human tissues and various animal models. For example, DβH versus tyrosine hydroxylase was visualized using differential staining in the human hindbrain while investigating catecholamines [13]. In addition, the determination of expression of DβH in the locus coeruleus of neonate rats after exposure to a commonly used herbicide was similar in methodology to the current study [14]. The studies just mentioned used immunostaining techniques that are similar to what was used in this study but were conducted on nervous tissue. The technique was expanded to include innervated tissues. More recently, this type of immunofluorescence has been used to investigate DβH colocalized in innervated tissues with neural markers, including PACAP. Their goal was to observe changes in neural markers in the ovaries of women suffering from polycystic ovary syndrome [15]. Those same immunofluorescence techniques were used in this study of innervated hepatic tissue.

As can be seen with the DβH concentrations in this study, even legally “safe” levels of BPA cause a statistically significant increase in DβH expression. Since DβH is mainly responsible for the synthesis of catecholamines, young vertebrates exposed to BPA will have increased levels of adrenaline in their liver and perhaps within their system even under normal nonstress conditions. This may lead to an increased fight or flight response, as well as problems with energy metabolism. Since the catecholamines epinephrine and norepinephrine increase heart rate and alertness, the elevated levels of DβH could possibly contribute to an increase in aggressive behavior, stress disorders, and increased rates of ADHD that have been previously correlated with BPA exposure in children, as described by several research teams [16,17,18,19]. Furthermore, increased catecholamines tend to change the normal functioning of energy metabolism by primarily affecting digestion and glucose levels. Therefore, it is possible that the increased levels of hepatic DβH may be behind the correlations of childhood obesity and diabetes that have been described by other research teams [20,21]. Although these are correlations found in children, they will most likely have life-long implications. ADHD and increased aggressive behavior will be a detriment to childhood education, for example, which may negatively impact the standard of living later in life. Increased BMI and diabetes usually affect individuals throughout their entire life and are known to reduce life expectancy in adulthood. However, future research would need to be performed in order to verify that these negative trends persist into later life. As a marker for the SNS, DβH shows signs that catecholamines are elevated in innervated hepatic tissue after BPA exposure, even at levels that are currently considered to be safe in humans. However, this study also colocalized DβH with another neuronal “gut–brain” marker, PACAP. DβH shows that the effects occur in the SNS, while the PACAP marker indicates a change in specific neurochemical coding.

The marker that was colocalized with DβH was PACAP. PACAP has been known to increase the amount of cAMP within cells, which is an important signaling molecule in many cellular pathways. PACAP has also been shown to increase the activity of the pituitary gland, and it acts in a similar way to vasoactive intestinal peptide (VIP), which is often expressed in the PNS [6,22]. Increased levels of PACAP have been associated with PTSD and other mental disorders in a well-known article published in the journal *Nature* [23]. Our results show that the level of DβH colocalized with PACAP increases dramatically at recommended safe levels and then remains roughly at the same level even at higher bisphenol exposure levels. At suggested safe levels, colocalized PACAP+/DβH+ is 166.7% higher when compared to controls, while at 10 times the safe exposure level, colocalized PACAP+/DβH+ is 135.6% higher than the control samples. It is safe to assume that BPA at recommended safe levels is already causing the maximum amount of PACAP+ nerve fibers, which is nearly three times higher when compared to the control samples. If these increases in colocalized nerve fibers are also occurring in the developing human population, the drastically increased PACAP+/DβH+ nerve fibers could be indicative of increased behavioral and psychological disorders in children and young adults, such as increased aggressive behavior, ADHD, and other psychological disorders. In order to confirm such a hypothesis, many more studies would need to be conducted; however, it appears to be more than just a possibility. The correlation between these psychological effects and BPA exposure has been described by numerous authors in various studies [16,17,19,24]. These studies have all positively correlated increased urine or plasma BPA levels with increased behavioral disorders in children and adolescents. Since dietary toxins come into contact with the liver before other organ systems, the effects may be more pronounced than in other innervated tissues. However, PACAP expression at nearly 3 times normal levels in the liver is indicative of increased expression throughout the entire neuronal system as well as in most other innervated tissues. Therefore, further studies are required to confirm or contradict that PACAP+/DβH+ nerve fibers are also elevated in other nervous systems of the developing vertebrate after BPA exposure.

It is very possible for a developing child to become exposed to at least 10 times the legal safe amount of BPA, since it is found everywhere in the polluted world and many parents may still be unaware of the dangers of BPA exposure, especially in developing countries [25]. Therefore, it is recommended to perhaps revise the current suggested safe levels of BPA exposure downward, as well as increase the awareness of the public to the potential dangers of BPA—especially with regard to children.

## 5. Conclusions

This study aimed to analyze the effects of a very common EDC (BPA) on the nerve fibers of the developing liver, as compared to a control group. The correlation effects seem to be mainly observed in human children, even though those effects may not be limited to children only. Therefore, this study observed nerve fibers in the SNS portion of the autonomic nervous system of the developing porcine liver after exposure to varying levels of BPA and compared the results to an identical control group with no BPA exposure.

The number of DβH nerve fibers was significantly increased, which indicates an overly active SNS. This seems to be evidence that bisphenol exposure at recommended safe levels is still too high, especially for children. It is hoped that this study may be an early step in determining if BPA exposure is upregulating the SNS as well as PACAP nerve fibers which may lead to pathological disease states that have already been correlated with bisphenol exposure. More studies should be performed using these neuronal markers to test other innervated tissues as well as the central nervous system and peripheral nervous systems. Using innervated hepatic tissue to test ingested BPA exposure is logical, since the liver is the first major organ exposed to dietary toxins. By testing increased or decreased colocalization in the liver, we have shown that BPA is causing significant changes. Hopefully, in a relatively short time, we may have a more detailed picture of how BPA is affecting the various nervous systems during child development.

## Figures and Tables

**Table 1:** Detailed list of primary and secondary antibodies used in the study.

**Primary Antibodies**				
Antisera	Code	Host Species	Dilution	Supplier
DβH	MAB 308	mouse	1:1000	Chemicon International Inc., UK; www.chemicon.com, 12 June 2019
PACAP	IHC 8922	rabbit	1:20,000	Bachem AG; www.bachem.com, 12 June 2019
**Secondary Antibodies**				
Reagent			Dilution	Supplier
Donkey anti-mouse IgG (H+L) conjugated with FITC			1:800	715-095-151; Jackson IR Lab, US; www.jacksonimmuno.com, 12 June 2019
Biotinylated goat anti-rabbit immunoglobulins			1:1000	E0432, DAKO Corporation, US, www.dakousa.com, 12 June 2019
Biotin-conjugated F(ab)’ fragment of affinity purified anti-rabbit IgG (H+L)			1:1000	711-1622, BioTrend, Germany; www.biotrend.com, 12 June 2019
CY3-conjugated Streptavidin			1:9000	016-160-084, Jackson IR Lab, US; www.jacksonimmuno.com, 12 June 2019

**Table 2:** Changes in the count of intrahepatic sympathetic nerve fibers as well as altered neurochemical characteristics after exposure to BPA under physiological conditions.

Neurochemical Characteristic	Groups of Animals		
	CTRL	E1	E2
DβH+	21.2 ± 2.08	31.5 ± 1.85	34.7 ± 1.61
DβH+/PACAP+	9.0 ± 2.07	24.0 ± 3.16	21.2 ± 3.81

Studied groups of animals: C—control; E1—experimental group 1 (low dose of bisphenol A); E2—experimental group 2 (high dose of bisphenol A). All results are statistically significant.

## Data Availability

Data sharing not applicable.

## Abbreviations

ANS	autonomic nervous system
BPA	bisphenol A
DβH	dopamine beta-hydroxylase
EDC	endocrine-disrupting compound
PACAP	pituitary adenylate cyclase-activating polypeptide
PSNS	parasympathetic nervous system
SNS	sympathetic nervous system
VIP	vasoactive intestinal peptide
